# What can we learn from sonication results of breast implants?

**DOI:** 10.1371/journal.pone.0182267

**Published:** 2017-08-10

**Authors:** Frederike M. J. Reischies, Robert Krause, Judith Holzer, Fabian Tiefenbacher, Raimund Winter, Gertraud Eylert, Tobias Meikl, Alexandru Tuca, Martin J. Köfer, Lars P. Kamolz, David B. Lumenta

**Affiliations:** 1 Department of Surgery, Division of Plastic, Aesthetic and Reconstructive Surgery, Medical University of Graz, Graz, Austria; 2 Department of Internal Medicine, Section of Infectious Diseases and Tropical Medicine, Medical University of Graz, Graz, Austria; 3 Department of Surgery, Landeskrankenhaus Feldbach/Fürstenfeld, Feldbach, Austria; 4 Institute for Hospital Hygiene and Microbiology, Medical University of Graz, Graz, Austria; University of South Australia, AUSTRALIA

## Abstract

**Background:**

Different research groups have identified microorganisms on breast implants by sonication with significant correlation to the rate of capsular contracture. This substantiated the hypothesis of an infectious etiology of capsular contracture. However, no clinical consequence has been drawn from these results yet. Aim of this study was to review sonication results from breast implants and to evaluate the current preoperative antibiotic regime for breast-implant surgery.

**Methods:**

We compared breast implant sonication culture results from published reports and our own database. Current perioperative antibiotic recommendations were compared with the susceptibility profile of the found organisms.

**Results:**

We found Coagulase-negative staphylococci and Propionibacteria to be the main group of microorganism found by sonication on explanted breast implants. Most guidelines recommend cephalosporins for preoperative antibiotical prophylaxis for breast-implant surgery.

**Conclusion:**

There is a discrepancy between antibiotic activity of commonly used antibiotics for preoperative prophylaxis of surgical site infections, and microorganisms found by sonication on breast implants, suspected to trigger the formation of capsular contracture. A targeted antibiotic prophylaxis for breast implant surgery with glycopeptides (e.g. Vancomycin) should be considered for the prevention of capsular contracture.

## Introduction

Periprosthetic capsular contracture is one of the leading long term complications following reconstructive or aesthetic breast implant surgery. It has a reported incidence of 5–22% in the literature[1], depending on follow-up time and surgical indication for breast implantation. Capsular contracture of Baker grade 3 and 4[2] is a severe complication given the need for surgical revision due to malposition and pain. The etiology of capsular contracture, it´s treatment and prevention are not entirely understood. Many possible causative factors were discussed, e.g. immune mechanisms in foreign body reactions, postoperative haematoma or individual predisposition[1]. However, evidence-based support for these theories is lacking.

A chronic subclinical Infection, caused by biofilm forming bacteria (e.g. Coagulase-negative staphylococci (CNS) or *Propionibacterium spp*.) has already been suspected decades ago to play a role in the etiology of capsular contracture[3]. This hypothesis was supported by several studies, showing correlations between antiinfective measures preventing contamination of breast implants and lower rates of capsular contracture[4][5][6]. However, for a long time the consistent detection of microorganisms on explanted breast implant surfaces and it´s association to capsular contracture was not evident, possibly due to the lack of sensitivity of conventional culture methods.

Sonication of medical devices has been introduced as a valid tool to detect biofilms on various implant surfaces and to identify organisms by culturing the sonication fluid[7]. Implants are put into a bath, to which ultrasound is applied, breaking down any possible biofilm. This technique has significantly improved the detection of implant associated infections in orthopedic surgery[8]. Sonication of breast implants eventually allowed for the consistent detection of the same microorganism and significant correlation to capsular contracture[9][10][11], which strongly supports an infectious etiology of capsular contracture.

The use of systemic antibiotics for the prevention of capsular contracture has only a few proponents in the surgical literature, which reported no effect on the rate of capsular contracture using cephalosporins or penicillins [12][13]. 97% of plastic surgeons stated to use cefazolin as antibiotic prophylaxis for breast implantation surgery in breast reconstruction in a recent study[14]. Given that the detection and identification of microorganism on breast implants was reported by different groups with significant association between capsular contracture and sonication result, a more targeted antibiotic regimen for the prevention of capsular contracture should be considered.

To the best of our knowledge, this is the first paper comparing sonication results from different sources including our own data. Aim of this study was to evaluate the suitability of commonly used antibiotic prophylaxis against microorganisms detected by sonication of breast implants.

## Methods

After revision of the proposed study protocol with a waiver for additional informed consent and final approval by the local ethical board (Medical University of Graz, vote # EK 28–626 ex 15/16), we retrospectively analyzed breast implant sonication data from otherwise asymptomatic patients, aged 18 years and above, who were carriers of uni- or bilateral breast implants and had an elective implant removal at the University Hospital Graz, Graz, Austria, between January 2015 and April 2016. Patients with signs of local infection in the breast region (e.g. redness, pain, swelling etc.) or systemic infection (e.g. elevated temperature, elevated leucocyte count, elevated c-reactive protein) were excluded. We compared these breast implant sonication results to sonication data of previously published papers. Studies on sonication for breast implants were reviewed, and compared for all retrieved data points.

Current recommendations for perioperative antibiotic prophylaxis for breast implant surgery were extracted from published guidelines, compared to actual antibiotic susceptibility of organisms found in sonication specimens, and profiled for their suitability in perioperative prophylaxis for surgery involving breast implants.

### Patient data collection

Following ethical board approval, the following patient data were extracted from electronic patient records (SAP R/3, SAP Germany, Walldorf, Germany): Patient age, sex, reason for implantation, reason for explantation, indwelling time of implants, placement of implants, degree of capsular contracture, sonication culture result.

Degree of capsular contracture was classified according to Baker[2]: grade IV—hard, painful contraction, contracted capsule visible, distorted shape or/and placement; grade III—breast harder than normal, implant visible and palpable, distortion of the implant; grade II—implant palpable, implant not visible, increased firmness of breast; grade I—normal soft breast.

### Breast implant: Sonication and culture

Immediately after removal, implants were put in sterile 1000ml containers (Pathopack, Intelsius, York, UK) by the surgeon and covered with 500ml of sterile Ringer solution. The containers were transported to the Institute of Clinical Hygiene and Microbiology, Medical University of Graz for sonication analysis. The closed container was fist vortexed for 30 seconds at maximum speed. The container was then placed into the ultrasound bath and was sonicated for 5 minutes at a frequency of 40 kHz and 200 Watt power. The container was then again vortexed for 30 seconds to distribute detached biofilm components in the fluid homogeneously. The container was next opened in a laminar air-flow biosafety cabinet, to avoid contamination, and 20 ml was removed from the fluid. 10 ml were transferred in to an aerobic blood culture bottle and 10 ml were transferred into an anaerobic blood culture bottle. Blood culture bottles were incubated at 37°C for 7 days. Conventional microbiological techniques were used to identify positive cultures.

### Comparing results from other sonication publications

A systematic review of the literature was performed to identify studies that used sonication analysis for explanted breast implants. For identification of studies, we used the following search terms and search terms combination in the PubMed database: ("breast implants" OR "breast implant") AND ("sonication" OR "broth culture") AND ("capsular contracture" OR "capsular fibrosis"). Inclusion criteria for studies were as follows: Full text articles on breast implants explanted and swiftly analyzed by sonication without further processing. Exclusion criteria were: duplicate publication of data. Articles were included on the 15.12.2016. The systematic review was conducted by FMJR and JH. From included studies demographical data, clinical data as well as sonication results were copied from demographical and clinical data tables from full text articles (Table 1 and S1 Table) Case reports were excluded, and no further restriction on levels of evidence below that was applied. Accumulated data was compared using descriptive statistics.

**Table 1:** Microorganisms found by sonication of breast implants.

	Reischies et al 2017	Pajkos et al. 2003	Del Pozo et al. 2009	Rieger et al. 2013
Number of implants	28	27 (100%)	45	121
Number of implants excluded due to infection	6	0	0	9
Number of implants included	22	27	45	112
**Degree of capsule contraction**				
Baker 1–2	1/22 (4.5%)	8/27 (29.6%)	18/ 45 (40%)	22/89 (24%)
Baker 3–4	21/22 (95.5%)	19/27 (70.4%)	27/45 (60%)	68/89 (76%)
**Sonication results**		capsules n = 27		
Culture-positive	19/22 (86.4%)	18/27 (66.6%)	10/45 (22.3%)	40/89 (45%)
Culture-negative	3/22 (13.6%)	9/27 (33.3%)	35/45 (77.7%)	49/89 (55%)
Coagulase-negative staphylococci	18/19 (94.7%)	15/18 (83.3%)	5/10 (50%)	16 /40 (40%)
Propionibacterium spp	1/19 (5.3%)	2/18 (11.1%)	5/10 (50%)	18/40 (45%)
Bacillus spp.	0	1/18 (5.5%)	0	3/40 (7.5%)
Others	0	0	0	3/40 (7.5%)

**Table 1**, showing sonication results of breast implants and capsules from different publications: Pajkos 2003, Del Pozo 2009, Rieger 2013 and Reischies 2017. Coagulase-negative staphylococci and Propionibacteria were most often found.

### Review of antibiotic agents recommended by guidelines for prophylactic use in surgery to prevent surgical site infection

Current guidelines for prevention of surgical side infection were collected and, if available, antibiotic recommendations specifically for breast implant surgery were reviewed. Recommended antibiotical agents and microorganisms found by sonication analysis were evaluated for antibiotic suitability.

## Results

We included 28 implants from 20 patients, all females, into our study of which 6 implants (21.4%) were excluded because of clinical infection (pain, redness, swelling of breast). Mean age at explantation was 48 (range 19–75) years.

22 implants were included into further analysis, of which 16 (72.7%) were implanted for aesthetic reasons, and 6 (27.3%) for reconstructive reasons. Mean implant indwelling time was 16.7 (1–34.4) years. Data on location of implants was available for 16 of 22 implants (72.7%), of which 10 (45.5%) were positioned subglandular, 5 (22.7%) subpectoral and 1 (4.5%) was positioned underneath a latissimus dorsi muscle flap. Implant surface details were available for 19 of 22 implants (86.4%), of which 13 (68.4%) were textured, 3 (15.8%) were smooth, and 2 (10.5%) had a polyurethane surface.

The main reason for explantation was capsular contracture, with 21 (95.5%) of 22 implants, of which 7 implants were also ruptured. 1 (4.5%) implant was explanted due to patient request. Of 22 analyzed implants 1 (4.5%) was explanted from a breast, showing no sign of capsular contracture (Baker grade 1) and 21 (95.5%) implants came from breasts, which showed considerable capsular contracture, with 17 (80.9%) implants from breasts classified Baker grade 3, and 4 (19%) implants from breasts classified Baker 4.

### Sonication results

Culture results after sonication showed that out of 22 analyzed implants, 3 (13.6%) were culture negative, and 19 (86.4%) were culture positive, of which 1 (5.3%) with *Propionibacterium avidum* and 18 (81.8%) with Coagulase-negative staphylococci (CNS). 18 of 21 (85.7%) implants, which were explanted from breasts with capsular contracture (Baker 3/4) had positive sonication results. Since only one implant was explanted from a breast without signs of capsular contracture, a correlation analysis of positive culture results and rate of capsular contracture was not reasonable.

### Sonication results from previously published paper

Seven studies matched our initial search, after further screening five papers were found reporting of breast implant sonication results, of which all were published after 2000, and two excluded from further analysis due to overlapping patient cohorts[15][16], (Table 1).

Pajkos et al analyzed 21 implants and 27 capsule-pieces from 16 patients. Capsule material and, if available, implant material was analyzed after maceration by sonication. Of all sonication results in this study n = 48, 24(50%) were positive, of which 18/24 (75%) grew CNS, with significant association of CNS positivity and the presence of Baker grade 3/4[9]. Del Pozo analyzed 45 implants from 29 women. There was a significant difference between implants explanted due to capsular contraction and implants explanted for other reasons, regarding positivity of sonication culture results (p = 0.034). The main group of isolated bacteria found were CNS and *Propionibacterium spp*[10]. Rieger analyzed 121 breast implants from 84 patients of which nine were excluded because of clinical infection. There was a significant correlation between degree of capsular contracture and culture positivity after sonication. The main group of bacteria found were *Propionibacteria spp* and CNS[11].

### Antibiotic agents recommended by guidelines for perioperative prophylaxis

Despite some conflicting reports on antibiotic prophylaxis for breast implantation surgery[17][18], all reviewed guidelines recommended the use of antibiotic prophylaxis for breast implant surgery. The Sanford guide to antimicrobial therapy recommends cefazolin, 1-2mg iv single-shot preoperatively for breast surgery[19]. ASHP therapeutic guidelines recommend antibiotic prophylaxis for breast implantation surgery, as they count the implant as a risk factor for infection[20]. Also according to SIGN guidelines (updated 2014), in breast surgery involving implants for reconstructive or aesthetic reasons antibiotic prophylaxis is recommended[21]. Systemic reviews also recommend the use of antibiotic prophylaxis in breast implantation surgery[22][23]. In all reviewed guidelines cephalosporins are most often recommended.

These recommondations are congruent with the recent report of 97% of plastic surgerons using cefazolin for this type of surgery [14].

However, there is a discrepancy between optimal antibiotic efficacy of cephalosporines and microorganism found by sonication, which are high-profile suspects to trigger the formation of capsular contracture (Table 2).

**Table 2:** Antibiotic activity against different microorganism.

	Gram positive				Gram negative		
	Staph aureus MSSA	Staph aureus MRSA	Staph epidermidis CNS	Strep pneumoniae	E coli	Klebsiella spp	Pseudomonas aeruginosa
**Penicillins**							
**Benzylpenicillin**	**-**	**-**	**-**	**+**	**-**	**-**	**-**
**Ampicillin/Amoxicillin**	**-**	**-**	**-**	**+**	**v**	**v**	**-**
**Co-amoxiclav**	**+**	**-**	**-**	**+**	**+**	**+**	**-**
**Flucloxacillin**	**+**	**-**	**v**	**-**	**-**	**-**	**-**
**Cephalosporins**							
**Cefradine**	**+**	**-**	**v**	**+**	**+**	**v**	**-**
**Cefuroxime**	**+**	**-**	**v**	**+**	**+**	**+**	**-**
**Ceftriaxone**	**+**	**-**	**-**	**+**	**+**	**+**	**-**
**Ceftazidime**	**-**	**-**	**-**	**-**	**+**	**+**	**+**
**Macrolides/Lincosamides**							
**Erythromycin**	**+**	**v**	**-**	**+**	**-**	**-**	**-**
**Clarithromycin**	**+**	**v**	**-**	**+**	**-**	**-**	**-**
**Clindamycin**	**+**	**v**	**v**	**-**	**-**	**-**	**-**
**Aminoglycosides**							
**Gentamicin**	**+**	**+**	**v**	**-**	**+**	**+**	**+**
**Diaminopyrimidines**							
**Trimethoprim**	**v**	**v**	**-**	**-**	**+**	**+**	**-**
**Quinolones**							
**Ciprofloxacin**	**+**	**-**	**-**	**-**	**+**	**+**	**+**
**Levofloxacin**	**+**	**-**	**-**	**+**	**+**	**+**	**+**
**Glycopeptides**							
**Vancomycin IV**	**+**	**+**	**+**	**+**	**-**	**-**	**-**
**Teicoplanin**	**+**	**+**	**+**	**+**	**-**	**-**	**-**
**Nitroimidazoles**							
**Metronidazole**	**-**	**-**	**-**	**-**	**-**	**-**	**-**
**Tetracyclines**							
**Doxycycline**	**+**	**+**	**v**	**+**	**-**	**-**	**-**

**Table 2**, showing ineligibility of cephalosporins against staphylococcus epidermidis (coagulase negative staphylococci—CNS) and suitability of glycopeptide antibiotics against staphylococcus epidermidis (coagulase negative staphylococci—CNS). From „Antibiotic prophylaxis in Surgery”Scottish Intercollegiate Guideline Network, updated 2014.

„v”indicates variable antibiotic susceptibility according to local epidemiology.

## Discussion

The detection and identification of microorganism on breast implants by sonication, explanted from breasts with capsular contracture, substantiated the hypothesis of an infectious etiology of capsular contracture. The strong link between these microorganisms and capsular contracture was supported by Tamboto et al, who has demonstrated a causal link between subclinical infection, biofilm formation, and capsular contracture in a porcine model[24].

**Fig 1:**
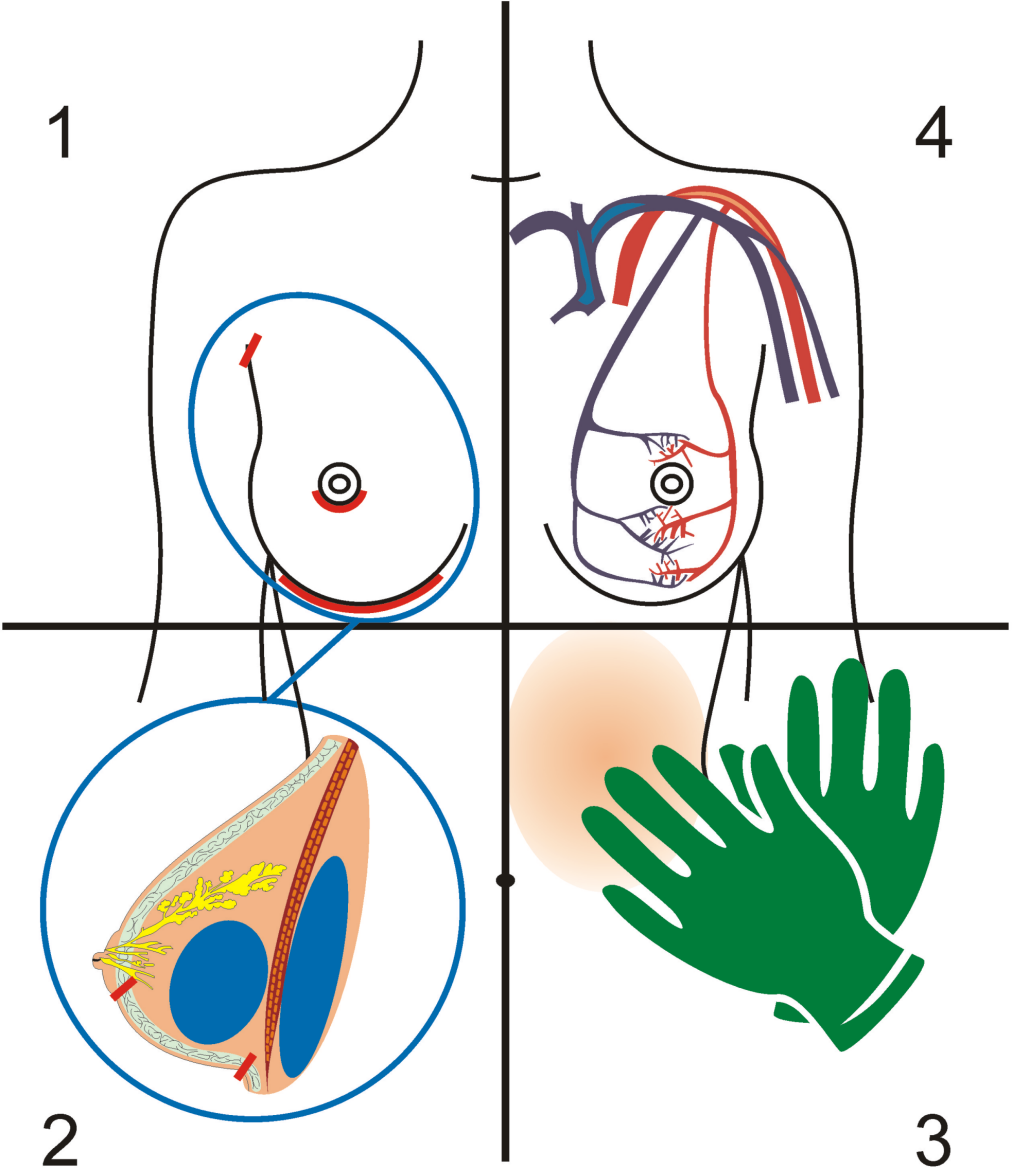
Contamination of breastimplants. Fig 1, showing possible sources of implant contamination; counterclockwise starting in the upper left corner (1) surgical access (marked in red: axillary, periareolar and inframammary), (2) implant contact with breast parenchyma (marked in blue: subglandular and infrapectoral implant placement), (3) contact with skin flora (patient, surgeron, scrup personnel), (4) haematogenous spread from asymptomatic bacteraemia.

Microorganism found by sonication form part of the skin flora, which are generally considered to have low virulence[25]. Fig 1 depicts possible sources of these microorganisms found on breast implants. Possible contamination of the implant during surgery may occur during contact with the skin flora (patient, surgeon, scrub personnel etc.)[26][27]. Contamination may also occur from microorganisms originating from breast ducts or glands, or result from asymptomatic bacteraemia of the patient[28][29].

Studies which reported methods to reach lower rates of capsular contracture describe methods to improve prevention of contamination of breast implants. Manual pocket dissection technique compared to diathermy dissection was reported to increase capsular contracture rates sevenfold in a study with 3002 patients[30]. A no-touch technique was proposed to reduce the risk of capsular contracture, since Mladick 1993 published a contracture rate of 0.6 percent in 2863 patients with a 17 year follow-up[5]. The implantation of suction drains were reported to increase the risk of capsular contracture more than fourfold[30]. Implants placed under the pectoral muscle have been reported to be associated with a decreased capsular contracture rate[31]. In the case of a periareolar implantation, the incidence of capsular contracture was 9.5 compared to 0.59 in the case of an inframammary access[6]. All of which presumably reduced the risk of contamination by the natural microbial flora of the breast ducts, by avoiding opening of breast gland ducts and allowing for a larger distance between breast gland and the implant. A systematic review showed no reproducible data regarding the association between type of implant surface and formation of capsular contracture[32]. The successful identification of microorganisms on breast implants with capsular contracture in recent years should be used to apply targeted antibiotic prophylaxis against the detected microorganisms for the prevention of capsular contracture.

Gylbert et al reported no significant difference in capsular contracture between two groups of patients, one receiving penicillin antibiotic prophylaxis perioperatively for breast implant surgery[12] These findings are in accordance with those of Mirzabeigi et al, who showed in a large cohort of primary and secondary breast augmentations, that three days of postoperative administration of cephalosporins did not not result in lower rates of complications such as infections or capsular contracture[33]. However, penicillin and cephalosporin antibiotics (beta-lactam antibiotics) do not provide suitable activity against microorganisms found on implants by sonication (Table 2)[34]. May et al reported 80% of CNS to be Oxacillin resistant in over 500.000 CNS isolates in the United States from 1999–2012, Oxacillin was tested as the antibiotic agent representative for beta-lactam antibiotics. Whereas, Vancomycin resistance in CNS isolates was not detected[34]. These results are in conjunction with reports from Asia and Europe where also very high rates of beta lactam antibiotic resistance (>70%–90%) in CNS isolates were reported[35][36]. We were unable to retrieve any other studies investigating systemic perioperative antibiotic prophylaxis for the prevention of capsular contracture.

**Fig 2:**
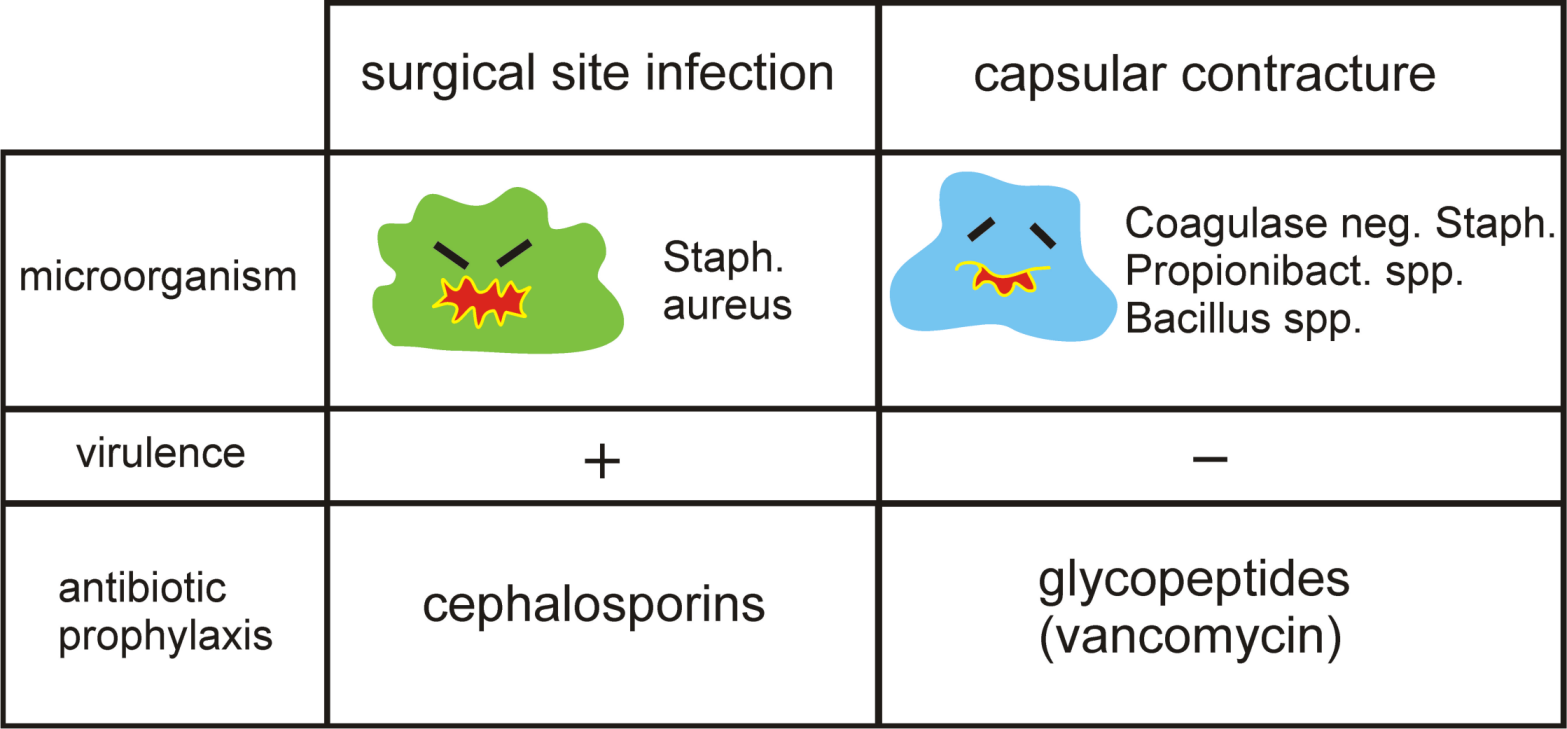
Capsular contracture vs surgical site infections. Fig 2, Comparing capsular contracture and surgical site infections, micoorganisms, virulence and antibiotic activity.

Although many different microorganisms can cause infections, surgical site infections are usually caused by a small number of common pathogens like *staphylococcus aureus* and *beta-haemolytic streptococci*. Antibiotic prophylaxis in surgery focuses on elimination of pathogens commonly responsible for surgical site infections (Fig 2). Microorganisms, which are suspected to trigger capsular contracture are considered to be of low virolence, thus they are not mainly targeted by currently recommended perioperative antibiotic regimens.

Therefore, cephalosporins, which are mostly used[14], do not provide sufficient activity against organisms isolated from breast implants by sonication, suspected to trigger the formation of capsular contracture. Glycopeptide antibiotics are highly efficient against gram positive organisms[34], like *propionibacteria spp* and CNS, the most frequently found microorganisms by sonication on breast implants[11][10][9]. As a result, for an efficient prevention of capsular contraction, glycopeptide antibiotics (Vancomycin) in addition to cephalosporins should be considered for preoperative antibiotic prophylaxis in breast implant surgery.

The benefit of using the proposed single iv dose of Vancomycin additionally to the single shot of cephalosporin, prior to breast implant surgery, results from the reliable antibiotic coverage against these microorganisms found by sonication on breast implants and the microorganisms typically responsible for surgical site infections. Vancomycin used alone as antibiotic prophylaxis led to higher rates of surgical site infections due to methicillin-sensitive staphylococcus aureus (MSSA)[37]. Actually Vancomycin plays a limited role in perioperative antibiotic prophylaxis, is only used in settings with high incidence rates of MRSA infections[38], and for patients with allergies against beta-lactam antibiotics[19]. As a side effect, Vancomycin has been reported to cause acute kidney injury and after fast infusion of Vancomycin a rash, that can affect the whole body, known as the red-man syndrome [39][40]. Therefore, patients considered for the proposed dual antibiotic regimen should be screened for kidney function prior to its administration and Vancomycin should be injected slowly, over 60 min prior to surgery.

Ideally biofilm formation and capsular contracture can be prevented by this targeted antibiotic prophylaxis of a cephalosporin in combination with Vancomycin, at a relatively low cost. One dosage of iv Vancomycin amounts to under 3 Euro at our institution and kidney function tests cost under 3 Euro. An acceptable trade-off, compared to the costs of possible revision surgery in the case of high grade capsular contracture, with resultant capsulectomy and bilateral implant exchange.

Breast implant operations have a considerably high share among aesthetic procedures, which is small compared to the overall number of surgical procedures in general. Therefore, the recommendation of a single iv dose of Vancomycin, interests only a negligible proportion of all surgical procedures, has a well-defined indication, and is based on our as well as previously published data [41]. Vancomycin resistance especially in enterococci emerged after the use of Avoparcin, a glycopeptid used as a growth promoter in food animals. Vancomycin resistant staphylococci in humans are still very rare despite Vancomycin being in place for almost 60 years [34][41].

Other antibiotic substances which can also be taken into consideration due to activity against CNS are Daptomycin and Trimethoprim/Sulfamethoxazole. Compared to Vancomycin, Daptomycin does not cause the red man syndrome (flush after injection) and shows no nephrotoxicity. However, one dose of Daptomycin costs about sixty times as much as one dose of Vancomycin. In our region Trimethoprim/Sulfamethoxazole has only weak activity against CNS with up to 30% resistance rates, and cannot be considered [35].

Local antibiotic solutions used during breast implant surgery such as local Gentamycin solution, do not provide reliable antibiotic activity against CNS, and cannot prevent the formation of a CNS biofilm around the implant–in our region resistance rates of CNS against Gentamycin reach up to 40%. There is also no official licensing for Gentamycin for local wound irrigation and the implants manufacturers do advice against the usage of any local wound solutions like betadine due to the possible interaction with the implant surface with unknown consequences.

Limitations include the retrospective design of our data review, and a possible non-exhaustive retrieval of additional publications (no records were identified through other sources on this topic as stated in the PRISM chart).

## Conclusion

Antibiotic single-shot intraoperative prophylaxis for breast implant surgery should be focused on eradication of CNS and propionibacteria, which seem to play a causative role in the formation of capsular contracture. Conventionally used cephalosporines alone for preoperative prophylaxis predominantly applied for prevention of surgical site infections are not suitable to eliminate organisms found to be associated with capsular contracture. We suggest to add Vancomycin to this regimen prior to breast implant surgery to target microorganisms found to be associated with capsular contracture, and aim to analyze the future outcome in a prospective fashion.

## Supporting information

S1 Table Additional information on sonication results. S1 Table, showing additional information on sonication results from following publications: Pajkos 2003, Del Pozo 2009, Rieger 2013 and Reischies 2017.
(DOCX)

S1 File Systematic Review PRISMA flow chart. (DOC)

S2 File Systematic Review PRISMA checklist. (DOC)
