# Supplementary material for: What can we learn from sonication results of breast implants?
Source: PLoS One. 2017 Aug 10;12(8):e0182267. doi: 10.1371/journal.pone.0182267 (PMC5552211; doi:10.1371/journal.pone.0182267)
Supplement: S1 Table — , showing additional information on sonication results from following publications: Pajkos 2003, Del Pozo 2009, Rieger 2013 and Reischies 2017. (DOCX) [file pone.0182267.s001.docx]

|  | **Reischies et al 2017** | **Pajkos et al. 2003** | **Del Pozo et al. 2009** | **Rieger et al. 2013** |
| --- | --- | --- | --- | --- |
|  |  |  |  |  |
| Number of implants | 28 | 27 (100%) | 45 | 121 |
| Number of implants excluded due to infection | 6 | 0 | 0 | 9 |
| Number of implants included | 22 | 27 | 45 | 112 |
| Age at explantation (yr) (mean [range]) | 48 (19-75) | 45.3 (29-56) | 61 (37-74) | 49 (19-80) |
| Reason for implantation |  |  |  |  |
| Aesthetic | 16 (72.7%) | 27 (100%) | 9/45 (20%) | 48(39.7%) |
| Reconstructive | 6 (27.3%) | 0 | 16/45 (35.5%) | 50 (41.3%) |
| other | 0 | 0 | 4/45 (8.8%) | 23(19%) |
| Reason for explantation |  |  |  |  |
| Capsule contration | 21/22 (95.5%) | n/a | 27/45 (60%) | 59 |
| additional impant failure | 7/21 (33.3%) | n/a | n/a | 8 |
| Patient request | 1/22 (4.5%) | n/a | n/a | 15 |
| Others/ no data | 0 | n/a | 18/45 (40%) | 30 |
|  |  |  |  | for 89 permanent implants: |
| Implant dwelling time (yr) (mean [range]) | 16.7 (1-34.4) | 9.2 (0.4-26) | 16.4 (0.65-33.9) | 4 (0.1-32) |
| location of Implant |  |  |  |  |
| Subglandular | 10/16 (45.5%) | 25/27 (92.6%) | n/a | 40/89 (44.9%) |
| Subpectoral | 5/16 (22.7%) | 3/27 (11.1%) | n/a | 41/89 (46.1%) |
| under flap | 1/16 (4.5%) |  | n/a | 0 |
| no data | 6/22 (27.3%) |  |  | 8/89 (8.9%) |
