# Supplementary material for: What can we learn from sonication results of breast implants?
Source: PLoS One. 2017 Aug 10;12(8):e0182267. doi: 10.1371/journal.pone.0182267 (PMC5552211; doi:10.1371/journal.pone.0182267)
Supplement: S1 File — (DOC) [file pone.0182267.s002.doc]

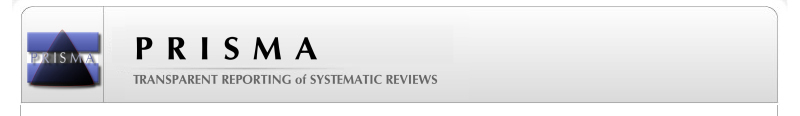
**PRISMA 2009 Flow Diagram**

**Screening**

**Included**

**Eligibility**

**Identification**

Records identified through database searching
(n = 7)

Additional records identified through other sources
(n = 0)

Records after duplicates removed
(n =7)

Records screened
(n = 7)

Records excluded
(n = 2)

Full-text articles assessed for eligibility
(n = 5)

Full-text articles excluded, with reasons
(n = 2)

Studies included in qualitative synthesis
(n = 3)

Studies included in quantitative synthesis (meta-analysis)
(n = 3)
